# Maternal Stress, Depression, Anxiety, and Participation in Care in Neonatal Semi-Intensive and Intensive Care Units: Results of a Cross-Sectional Study in Two Sri Lankan Hospitals

**DOI:** 10.3390/children13020247

**Published:** 2026-02-10

**Authors:** Nimesha Gamhewage, Mohamed Rishard, Nalin Gamaathige, Loshika Janet, Ilaria Mariani, Hemantha Senanayake, Marzia Lazzerini

**Affiliations:** 1 Faculty of Medical Sciences, University of Sri Jayewardenepura, Nugegoda 10250, Sri Lanka; 2 Colombo South Teaching Hospital, Kalubowila 10350, Sri Lanka; 3 Faculty of Medicine, University of Colombo, Colombo 00800, Sri Lanka; mrishard@obg.cmb.ac.lk (M.R.); losh.research2022@gmail.com (L.J.); senanayakeh@gmail.com (H.S.); 4 De Soysa Maternity Hospital for Women, Colombo 00800, Sri Lanka; 5 WHO Collaborating Centre for Maternal and Child Health, Institute for Maternal and Child Health—IRCCS Burlo Garofolo, 34137 Trieste, Italy; ilaria.mariani@burlo.trieste.it (I.M.); marzia.lazzerini@burlo.trieste.it (M.L.)

**Keywords:** mothers, stress, depression, anxiety, participation, NICU, Sri Lanka, LMIC

## Abstract

**Highlights:**

**What are the main findings?**

- We observed high psychological morbidity and limited maternal participation in newborn care among mothers of infants admitted to neonatal units.
- Anxiety and stress levels were higher among mothers who were less involved in the care of their neonates.

**What are the implications of the main findings?**

- Mothers should be encouraged to actively participate in the care of their newborns during neonatal unit admission.
- In Sri Lankan settings, routine screening for maternal psychological burden, along with policy reforms, is recommended.

**Abstract:**

**Background/Objectives**: Admission of a newborn to a neonatal intensive care unit (NICU) places mothers under considerable psychological strain, yet there is limited research from resource-limited settings regarding this aspect. This study represents the Sri Lankan arm of the multicentre study titled “Empowering Parents in the NICU”. It aimed to determine the prevalence and associated factors of stress, depression, and anxiety among mothers of neonates admitted to neonatal units and to assess mothers’ participation in neonatal care. **Methods**: A cross-sectional study was conducted in two tertiary neonatal units in Sri Lanka. Maternal stress, depression, and anxiety were measured using the Parental Stressor Scale: NICU, Edinburgh Postnatal Depression Scale, and State–Trait Anxiety Inventory. Maternal involvement in care was assessed using the Index of Parental Participation (IPP-NICU). Univariate and multivariate logistic regression analyses were performed. **Results**: A total of 300 mothers were enrolled. The prevalence of stress, depression, and state anxiety was 73%, 87%, and 77.7%, respectively. Overall, 94.3% experienced at least one psychological condition, while 59% experienced all three. Only 13% achieved an IPP-NICU score ≥ 20 (maximum: 30). Mothers of infants admitted to NICUs, compared with those in semi-intensive care, showed significantly higher rates of depression. Tamil and Muslim mothers demonstrated lower rates of state anxiety compared to Sinhalese mothers. An IPP-NICU score ≥ 20 was associated with reduced stress and anxiety. **Conclusions**: High psychological morbidity is observed among mothers of neonates managed in neonatal units, emphasising the need for routine maternal mental health screening and promoting maternal participation in neonatal care.

## 1. Introduction

Globally, up to 30 million neonates require inpatient care annually [1]. The reasons include prematurity, hypoxic ischaemic encephalopathy, neonatal sepsis, jaundice, and congenital anomalies [1]. The neonatal intensive care unit (NICU) environment is stressful for neonates, leading to detrimental effects on their health and development [2].

In recent years, there has been a growing recognition of the burden of parental stress, depression, and anxiety when a newborn is admitted to the NICU [3,4]. However, data from resource-poor settings are scarce. In a single-centre study conducted in a Sri Lankan NICU, 44% and 32% of mothers reported having severe and extreme levels of stress, respectively [5]. The primary causes of stress were changes in the parental role, the physical environment of the NICU, the severity of the baby’s illness, past neonatal fatalities, and having a previous baby who required NICU admission [5]. No data are available regarding the rates of depression and anxiety of mothers with a neonate admitted to the NICU in Sri Lanka.

Maternal stress, anxiety, and depression can lead to lasting psychological effects, necessitating support and intervention [6,7,8]. These negative experiences could adversely affect the developmental outcomes of infants. In addition, they lead to behavioural issues and affect parent-infant relationships and parental roles [7,9]. To mitigate these detrimental effects, interventions such as family-centred care and parental involvement in the care of newborns in the NICU are recommended [10,11]. However, awareness that parents can play a greater role in the care of their neonates in NICUs in resource-limited settings seems to be limited.

Sri Lanka has achieved remarkable gains in neonatal care over the past several decades. The neonatal mortality rate in 2023 was 7.2 per 1000 live births [12], which compares well with countries with a higher per capita income [13]. Despite these accomplishments, the psychological aspects have been neglected, and only a few studies have been conducted to evaluate parental psychological health [5], with none related to parental participation in their newborn’s care. In Sri Lankan NICU settings, the care is focused almost entirely on the outcomes of the neonates. Mothers are permitted to visit their infants only during designated times, and their involvement in neonatal care is largely limited to providing expressed breast milk and participating in kangaroo mother care.

This study was the Sri Lankan component of a multicentre international cross-sectional quality improvement project titled “Empowering parents in the NICU” (EPiNICU), which aimed to collect data and to develop and test evidence-based models of interventions to improve parental participation, mental health, and well-being of parents of newborns admitted to the NICU. This study was conducted to assess the prevalence and severity of NICU-related maternal stress, depression, and anxiety. We also assessed the level of maternal involvement in the newborn’s care while the baby was managed in the neonatal unit. Additionally, the study assessed risk factors associated with maternal psychological morbidity.

## 2. Materials and Methods

### 2.1. Study Design and Setting

Given the absence of prior data from Sri Lankan NICUs on these psychosocial outcomes, an observational cross-sectional design was necessary to characterise the local context. Therefore, this study was conducted in two tertiary care hospitals in Colombo, Sri Lanka, namely, the De Soysa Hospital for Women and the Colombo South Teaching Hospital. The two hospitals have a total of 10,000 deliveries per year and 1800 admissions to the neonatal units. The two units have 22 NICU beds and 20 semi-intensive care spaces. During the study period, 72 nurses, 27 doctors, and 4 neonatologists were in service in the two facilities. Both hospitals accept in utero transfers of complicated pregnancies and neonatal transfers. Results are reported according to the STROBE guidelines [14] (Supplemental Table S1). The study design was aligned with the EPINICU multicentre protocol to allow meaningful comparison across diverse healthcare settings.

### 2.2. Study Participants

Mothers having a neonate needing intensive care or semi-intensive care for more than 48 h at the study sites were eligible for inclusion. Mothers who were under 18 years of age, those with existing mental illness that precluded them from providing informed consent, and those facing the impending death of their baby were excluded.

A trained multilingual data collector approached the mothers close to the date of discharge from the neonatal unit and obtained informed written consent before recruitment. A convenience sampling method was followed based on the availability and willingness to participate of mothers. The data collector used the NICU and semi-intensive care unit registry. Mothers were approached in the waiting area of the neonatal unit, where they generally remain until accompanied by a staff member to the postnatal wards.

### 2.3. Data Collection Tools

#### 2.3.1. Parental Stressor Scale: NICU

The reduced version of the Parental Stressor Scale (PSS:NICU), a scale specific to measuring parental stress related to the NICU, was utilised to measure stress [15]. PSS:NICU has been used previously in Sri Lanka [5]. The scale is composed of 26 statements categorised into three domains: stress resulting from “sights and sounds”, “infant behaviour and appearance”, and “parental role alteration”. Questions in the PSS:NICU do not refer to a specific period of time. Stress is scored on a five-point Likert scale, with one point assigned for “not at all stressed” and five points assigned for “severe stress”. The total PSS:NICU scores are determined in two ways: the stress occurrence level (SOL) and the overall stress level (OSL). SOL is computed using only experienced items, and OSL with one point deducted for “not applicable” items. In this study, we determined only the SOL score, as it is suggested to use the OSL when the focus is on the NICU environment and the SOL when the focus is on the parent [15]. A cut-off of three or more has been used in the literature to identify those affected by stress [16].

#### 2.3.2. Edinburgh Postnatal Depression Scale

The Edinburgh Postnatal Depression Scale (EPDS), which has been validated in Sri Lanka, was utilised to assess the presence of symptoms of depression [17]. There are ten items on the scale, each with four answers, with scores ranging from 0 to 3. According to the total score obtained, the severity of symptoms is categorised as none or minimal depression (0–6), mild depression (7–13), moderate depression (14–19), and severe depression (20–30) [18]. Validated versions of this tool are available both in Sinhala and Tamil. The Sri Lankan study that validated the EPDS suggested a cut-off score of 9 for screening for depression in our settings [17].

#### 2.3.3. State–Trait Anxiety Inventory

Anxiety was evaluated by the State–Trait Anxiety Inventory (STAI), which is composed of two scales: state anxiety (Y1), i.e., how one feels at the moment, and trait anxiety (Y2), i.e., how one generally feels [19]. Each STAI scale contains 40 items with a four-point Likert scale (from 0 to 3 points). The questionnaire is not specific to parents’ anxiety in the NICU [20]. The presence of anxiety was identified with a score above 40. Mild, moderate, and severe anxiety are indicated by scores between 41 and 50, 51–60, and >60, respectively [20,21]. STAI has been previously validated and used in Sri Lanka [22]. We utilised state anxiety scores to describe maternal anxiety.

#### 2.3.4. Index of Parental Participation

Participation in care was assessed via the Index of Parental Participation (IPP-NICU) questionnaire. This was originally used on parents whose children were admitted to the hospital [23]. We used an adapted version of it to reflect activities related to newborn care. This adapted version has been successfully used in similar low-resource settings [24]. The IPP-NICU assesses parental involvement during the previous 24 h in four domains: activities related to daily living (6 items), providing comfort (7 items), advocating for newborn health (7 items), and technical tasks (10 items). There are no recommendations in the literature regarding the cut-off score of the IPP-NICU, but we used a cut-off of 20 in accordance with previous studies [16]. The maximum score is 30.

The study instruments, other than the EPDS and STAI, were translated and back-translated to Sinhala and Tamil, the two main languages spoken in the country, by two qualified translators to ensure accuracy and consistency.

Details of the baby (gestational age at birth, birth weight, APGAR score, complications during the hospital stay, need for intubation, surgical interventions, and length of stay) were collected from the patient records. Details regarding the mothers’ socioeconomic background and educational status were obtained directly from the interview with the mothers.

### 2.4. Data Collection Procedure

The eligible mothers were approached by a trained research assistant who was not involved in patient management or data analysis and was fluent in all three languages used in the country: Sinhala, Tamil, and English. All questionnaires were self-administered, and hard copies were provided to the participants. Data were collected from November 2023 to September 2024 at both sites. In January 2024 and May 2024, the data collector was not available. Therefore, mothers were not approached for enrolment in those months.

### 2.5. Study Outcomes and Sample Size Calculation

Prevalence of maternal NICU-related stress evaluated with SOL was predefined as the primary study outcome. Other key outcomes were the prevalence of maternal depression, maternal state and trait anxiety, and maternal participation in newborn care.

A sample of 271 mothers was required based on the reported prevalence of stress in parents of newborns in neonatal units of 50 ± 10% [16], with a confidence level of 99.9%.

### 2.6. Data Analysis

Descriptive statistics were used to describe the characteristics of neonates and mothers, to summarise the scores of depression, stress, anxiety, and parental participation in care, and to determine the prevalence of the above psychological conditions.

Univariate and multivariate logistic regression models were conducted for each mental health condition under analysis (i.e., depression, stress, and anxiety) considering the following variables as explanatory variables: maternal participation in care (dichotomised as IPP-NICU ≥ 20 or below), maternal socioeconomic background (ethnicity, maternal age, and maternal occupation), and newborn characteristics (APGAR at 5 min, the birth weight of the neonate, gestational age, need for ventilation, admission to NICU/semi-intensive care, presence of respiratory distress, sepsis, and total duration of hospital stay). For twin pregnancies in which both infants required admission, only the characteristics of the first twin were collected and included in the analysis.

Dependent variables were analysed as binary variables using the following cut-offs: SOL ≥ 3, EPDS ≥ 9, and STAI Y1 > 40. Odds ratios (ORs) and adjusted odds ratios (aORs) were calculated, with 95% confidence intervals (CIs) and *p* values of significance.

All statistical tests were two-tailed. Statistical analyses were performed using Stata V.14 and R V.4.1.1. A *p*-value less than 0.05 was considered statistically significant.

## 3. Results

**Figure 1:**
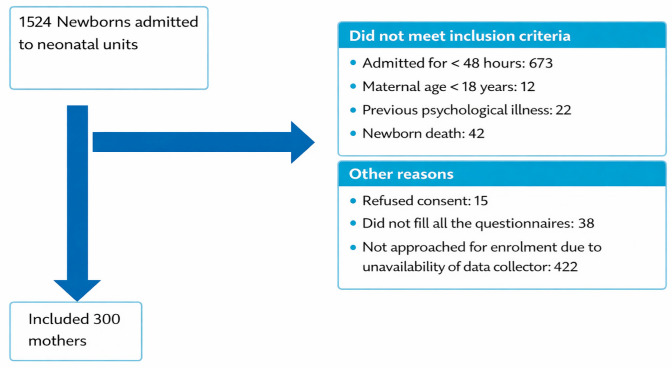
Study flow diagram.

Among the 1524 neonates admitted to neonatal units at the study sites during the study period, 775 were eligible. Very few mothers refused participation (*n* = 15). Overall, we included 300 neonates and 300 mothers (Figure 1).

The characteristics of the mothers are summarised in Table 1. The median age of mothers was 30 years (IQR 26–34, mean 30, SD 6). All mothers were married, and most belonged to the Sinhala ethnic group (*n* = 199, 66.3%). All had received either secondary or higher education.

The characteristics of the neonates are summarised in Table 2. The median birth weight was 2335 g (IQR 1430–2990, mean 2238.2, SD 885.7). Approximately a quarter of newborns were born less than 32 completed weeks of gestation, and 27.6% (*n* = 83) weighed less than 1500 g. The majority were admitted to the NICU (78.3%).

### 3.1. Overall Prevalence of Stress, Depression, and Anxiety

**Figure 2:**
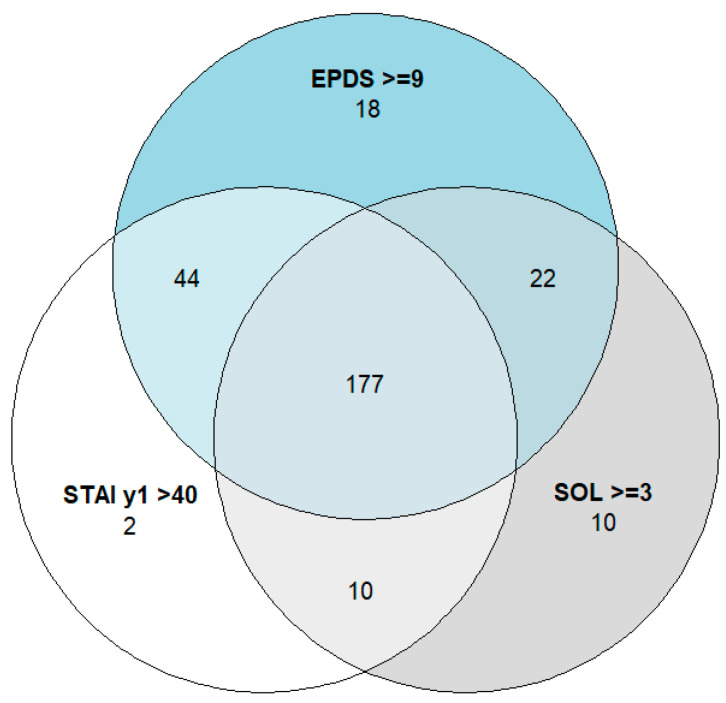
The pattern of overlap among conditions. (Abbreviations: EPDS—Edinburgh Postnatal Depression Scale; SOL—stress occurrence level; STAI y1—State Anxiety Scale. The following cut-off values were used: SOL ≥ 3, EPDS ≥ 9, and STAI Y1 > 40). Notes: In total, 16 mothers have no condition, and PSS:NICU was not collected from one mother.

A total of 283 (94.3%) mothers were detected as experiencing at least one condition, whereas 177 (59.0%) experienced all three conditions together (Figure 2). In the study sample of 300 mothers, only 16 reported no condition. The PSS:NICU score was not collected for one mother.

### 3.2. Prevalence of Stress, Depression, and Anxiety

#### 3.2.1. Maternal Stress

**Figure 3:**
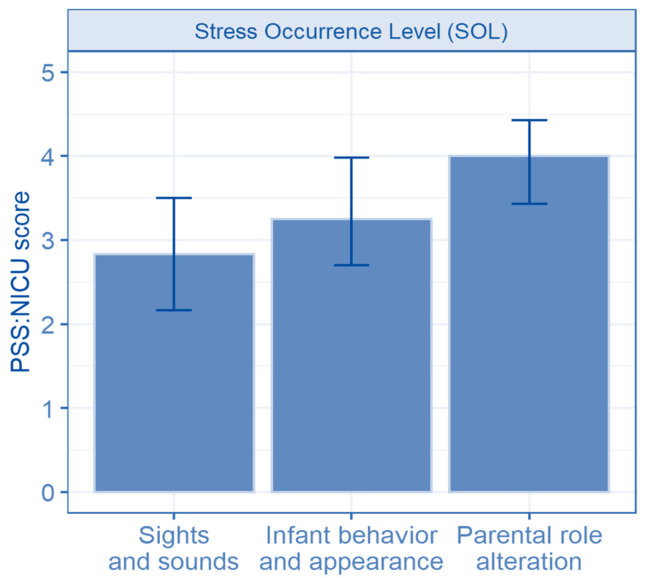
Stress by domain—median scores and IQR (Abbreviations: PSS:NICU score—Parental Stressor Scale NICU score).

A total of 219 mothers (73%) scored 3 or more on the PSS:NICU SOL score, indicating the presence of stress. The mean PSS:NICU SOL score was 3.34 (Table 3). The highest mean subscore was noted in the parental role alteration domain (mean 3.81, SD 0.89; median 4, IQR 3.43–4.43) (Figure 3, Supplemental Table S2).

#### 3.2.2. Maternal Depression

**Figure 4:**
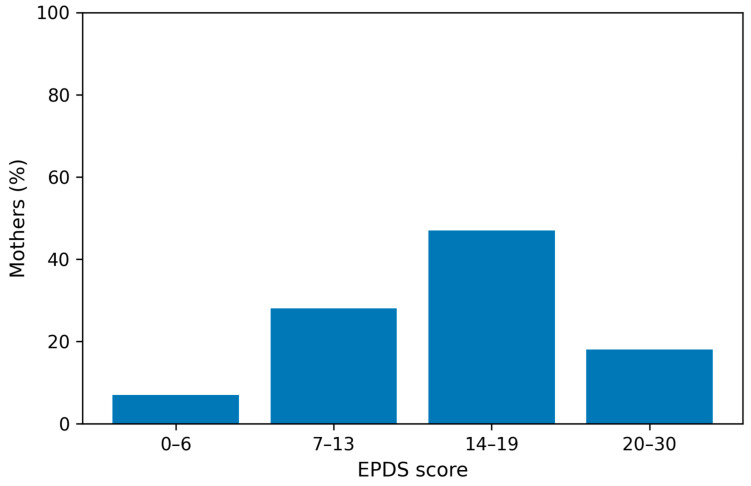
Severity categories for depression. (Abbreviations: EPDS—Edinburgh Postnatal Depression Scale). Notes: Percentages are calculated on the sample of 300 mothers.

A total of 261 mothers (87%) scored 9 or more in the EPDS score (Figure 2). The median EPDS score for the study population was 15 (IQR 11–18, mean 14.76, SD 5.4) (Table 3). In total, 140 mothers (46.7%) scored 14–19 (moderate symptoms), while 53 mothers (17.6%) scored 20–30 (severe symptoms) in the EPDS score (Supplemental Table S3). Figure 4 illustrates the severity categories of depression.

#### 3.2.3. Maternal Anxiety

The mean STAI state score was 45.52 (SD 8.42; median score 46, IQR 41.50–50.50), and 77.7% of mothers (*n* = 233) had scores above 40 (Table 3, Figure 2). The majority of mothers (*n* = 158, 52.7%) had mild anxiety symptoms (Supplemental Table S3).

Trait anxiety was present in 96.7% (*n* = 290) of mothers (mean score 45.79, SD 3.23; median 47, IQR 45–48) (Table 3). The majority of mothers *(n* = 268, 89.3%) had mild levels of trait anxiety (Supplemental Table S3).

**Figure 5:**
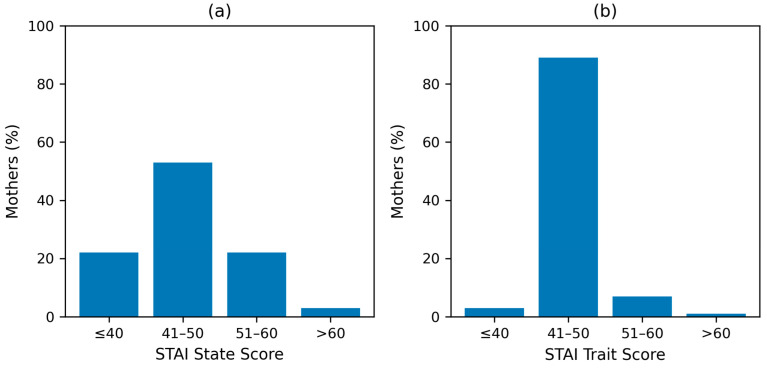
Maternal state and trait anxiety: severity categories. (**a**) STAI state score categories. (**b**) STAI trait score categories. (Abbreviations: STAI = State–Trait Anxiety Inventory). Notes: Percentages are calculated on the sample of 300 mothers.

Figure 5 illustrates the severity categories of maternal state and trait anxiety.

### 3.3. Maternal Participation in Care of the Neonate

**Figure 6:**
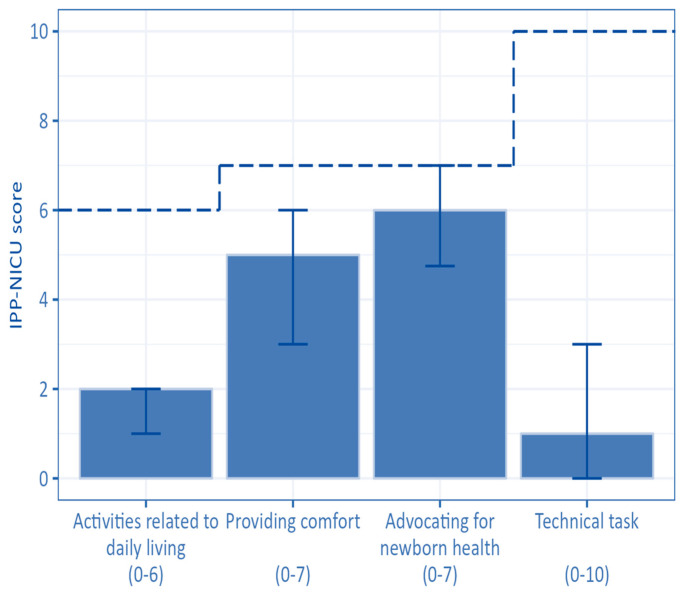
Parental participation, by domain: median scores and IQR. (Abbreviations: IPP-NICU score—Index of Parental Participation in Neonatal Intensive Care score). The dotted line represents the maximum attainable score, which is also reported in brackets for each domain on the x-axis.

The mean IPP-NICU score was 13.81 (SD 4.95; median score 13, IQR 11.00–16.50) (Table 3). Only 13.0% of mothers (*n* = 39) had IPP-NICU scores ≥ 20. The domain with the lowest median value was the “technical tasks” domain (mean 2.03; SD 2.72; median score 1, IQR 0–3). The median scores and the IQR of the IPP-NICU are shown in Figure 6 (Supplemental Table S4).

### 3.4. Logistic Regressions

#### 3.4.1. Stress

Univariate analysis revealed a statistically significant association between lower rates of stress in Tamil mothers compared to Sinhalese mothers (OR 0.37, 95% CI 0.19–0.73, *p* = 0.004), and mothers with IPP-NICU scores ≥ 20 (OR 0.25, 95% CI 0.12–0.50, *p* < 0.001). Multivariate analysis confirmed results for mothers with IPP-NICU scores ≥ 20 (aOR 0.27, 95% CI 0.12–0.59, *p* = 0.001) (Table 4).

#### 3.4.2. Depression

In univariate analysis, we found EPDS scores ≥ 9 more frequently in mothers with babies born at 32–37 weeks of gestation, compared with the group <27 weeks of gestation (OR 5.44, 95% CI 1.01–25.29, *p* = 0.034), and less frequently in babies admitted to the semi-intensive care unit compared to NICU admission (OR 0.43, 95% CI 0.21–0.91, *p* = 0.023). The multivariate analysis confirmed that the odds of depression were lower in mothers with babies admitted to a semi-intensive unit compared to the NICU (aOR 0.29, 95% CI 0.12–0.68, *p* = 0.004) (Table 5).

#### 3.4.3. Anxiety

Univariate analysis revealed that mothers with IPP-NICU scores ≥ 20 (OR 0.4, 95% CI 0.20–0.83, *p* = 0.011) and both Tamil and Muslim mothers compared to Sinhala mothers (Tamil: OR 0.35, 95% CI 0.17–0.72, *p* = 0.004; Muslim: OR 0.43, 95% CI 0.22–0.85, *p* = 0.014) had lower chances of having STAI state anxiety scores > 40. The multivariate analysis confirmed these results (Tamil: aOR 0.27, 95% CI 0.12–0.63, *p* = 0.002; Muslim: aOR 0.34, 95% CI 0.16–0.73, *p* = 0.005; IPP-NICU scores ≥ 20: aOR 0.32, 95% CI 0.14–0.72, *p* = 0.005) (Table 6).

## 4. Discussion

This is the first comprehensive study from a South Asian setting to report maternal psychological morbidity when a baby is admitted to neonatal units. The findings are generally in concordance with other settings, but we found the prevalence of stress, anxiety, and symptoms of depression to be much higher in our study [3,24,25].

Symptoms of depression were found in 87.0% of the study participants. Other studies have reported a prevalence of 18.0% and 52.0% [3]. A multicentre study involving Italy, Brazil, and Tanzania reported prevalences of 33.3%, 35.8%, and 52.3%, respectively [24]. A community-based study conducted in two Medical Officer of Health areas in Sri Lanka revealed a prevalence of 15.5% on the 10th postpartum day and 7.8% at 4 weeks postpartum [26].

Compared with mothers whose babies required admission to the semi-intensive care unit, those whose babies were admitted to the NICU had a significantly greater prevalence of depressive symptoms. This indicates a need for routine screening of mothers for depression when babies require admission to the NICU. This finding aligns with recommendations for the routine screening of mood and anxiety disorders by many authorities [27,28]. As in several other settings, we could not observe a significant association between maternal sociodemographic characteristics, complications of the infant, or the total duration of hospital stay [24,25]. However, prematurity has been identified in the literature as a potential risk factor for postpartum depression [29]. In our study, the lack of an observed association in multivariate regression between prematurity and maternal depression may be attributable to the small sample size of mothers without depressive symptoms. Notably, the EPDS is only a screening tool, and confirmation of the diagnosis requires clinical input [30].

Similar to a previous study conducted in Sri Lanka regarding maternal stress [5], our study revealed a high prevalence of stress (74%). Furthermore, the observed prevalence was higher than that reported from other EPiNICU project facilities, where rates ranged from 4.5% in Tanzania to 64.5% in Brazil and 36.7% in Italy [24]. The domain with the highest levels of stress was the alteration in the parental role, similar to the findings of previous studies [16,24]. Mothers often feel unprepared for discharge due to limited engagement with their neonates, which can lead to emotional strain and increased risk of readmission [31]. This is further supported by the association of a higher incidence of stress in mothers who were less involved in their baby’s care (IPP-NICU scores less than 20). These factors highlight the need to guide and encourage mothers to play a greater role in their baby’s care. Variables such as maternal age, duration of stay in the NICU, the presence of neonatal seizures, and the need for ventilator support have been described as factors associated with increased stress [32]. However, in our study, no association was observed between these factors and maternal stress, indicating that the determinants of maternal stress may differ across settings.

We found a higher prevalence of state anxiety (77.7%) than in other facilities participating in the EPiNICU project (36.9% in Italy and 59.3% in Brazil) [24]. Sinhalese mothers were more likely to report state anxiety than the other two ethnic groups. Anxiety related to ethnicity has been reported in previous studies as well [24]. This may be an aspect that is worthy of further study. The associations between low IPP-NICU scores and anxiety and stress are significant findings that have not been demonstrated in other countries [24].

The score for participation in care was lower in our study population than in other studies. Using the same instrument, parents in Tanzania, Italy, and Brazil scored median IPP-NICU scores of 24 (IQR 21; 26), 18 (IQR 12; 22), and 21 (IQR 15; 24.8), respectively [24]. Similar to our observations, no country reached the highest possible level of parental participation in care. Furthermore, the largest gap across all countries was found in the “Technical tasks” domain [24]. In Sri Lankan NICUs, maternal involvement in neonatal care is not actively encouraged. The results of our study emphasise the need for a change in the culture of Sri Lankan neonatal units, promoting structured support systems, such as family-integrated care, which promote parental involvement and empowerment in NICUs [11,33]. Participation in care is a modifiable factor that could be achieved with changes in NICU policies and could influence maternal stress and anxiety levels.

Beyond the risk factors assessed in this study, maternal chronic illnesses, particularly those affecting fertility such as endometriosis, can contribute to a significant mental health burden, which may be present even before conception and persist after delivery [34,35].

We acknowledge the following limitations of our study. First, as it is a cross-sectional study, causal inference among different study variables cannot be assessed. However, it highlights a problem that is often overlooked in NICU settings. Second, except for EPDS and STAI, other study instruments have not been validated in Sri Lanka. Nevertheless, these questionnaires have been widely used in different contexts, including LMICs. Furthermore, the findings of this study cannot be generalised to the whole country, as we conducted the study in two urban tertiary care hospitals. However, we were able to include study participants from various socioeconomic, educational, and ethnic backgrounds. In addition, exclusion of mothers younger than 18 years, who are considered a vulnerable population requiring additional ethical safeguards, may limit the generalisability of the findings to adolescent mothers with neonates admitted to the NICU. Finally, these findings indicate the need for further studies in similar settings and also to study the psychological burden of fathers.

## 5. Conclusions

Our study demonstrates an exceptionally high prevalence of depressive symptoms, anxiety, and stress among mothers of neonates admitted to Sri Lankan NICUs, which is a significant, previously under-recognised entity. The strong association between low maternal participation in neonatal care and increased psychological distress highlights the importance of supporting maternal involvement in the newborns’ care during NICU admissions. These findings provide context-specific evidence to inform improvements in neonatal care practices and policy development in Sri Lanka, in accordance with the objectives of the EPiNICU project, which aimed at identifying and developing context-specific interventions to empower parents in NICUs.

Routine screening for maternal psychological conditions using validated tools, such as the EPDS, should be integrated into standard NICU care to enable early identification and timely intervention. Collaboration with mental health services within NICUs should be promoted. Introducing FCC models to Sri Lankan NICUs may help reduce maternal psychological distress and empower parents. Culturally sensitive psychosocial support is essential, given the observed ethnic differences in anxiety levels.

Beyond the clinical setting, admission to a NICU should be recognised as a significant risk factor for maternal mental health problems. Mothers with hospitalised neonates should be included as a high-risk group in public health programmes. Early identification and support during the immediate postnatal period, alongside staff training in recognising maternal distress and implementing family-centred care, have the potential to improve maternal well-being, mother–infant bonding, and long-term child developmental outcomes.

## Figures and Tables

**Table 1:** Characteristics of mothers.

	Number	%
Maternal age		
18 to 29 years	147	49
30 to 39 years	137	45.6
>40 years	16	5.3
Occupation		
Employed	56	18.6
Marital status		
Married	300	100
Ethnicity		
Sinhala	199	66.3
Tamil	44	14.7
Muslim	57	19
Maternal educational status		
Secondary	18	6.0
Higher	282	94.0

Notes: Percentages are calculated on the sample of 300 mothers.

**Table 2:** Characteristics of neonates.

	Absolute Frequency (n)	Percentage (%)
Male	177	59.0
Multiple pregnancy *	19	6.3
APGAR at 5 min less than 7	5	1.6
Admitted to the NICU	235	78.3
Admitted to semi-intensive care unit	65	21.7
Ventilated **	200	66.7
Required surgery	11	3.7
Gestational Age		
<27 weeks	11	3.7
27–31 + 6 weeks	59	19.7
32–36 + 6 weeks	93	31.0
≥37 weeks	137	45.7
Birth weight		
<1000 g	25	8.3
1000–1499 g	58	19.3
1500–2499 g	85	28.3
≥2500 g	132	44.0
Number of days stayed in the hospital		
<7 days	56	18.6
7 days–30 days	186	62.0
31 days–90 days	53	17.6
>90 days	5	1.6
Complications of neonates		
Respiratory distress	210	70.0
Jaundice	37	12.3
Neurological event ***	22	7.3
Major birth trauma ****	3	1.0
Major congenital malformation *****	18	6.0
Sepsis ******	110	36.7

Notes: Percentages are calculated on the sample of 300 newborns. * There were 2 groups of triplets and 17 pairs of twins. Only one neonate from each triplet group required admission to the neonatal unit. Among the twins, only seven twin pairs needed admission of both neonates; we collected the characteristics of the first twin only. ** Defined as needing mechanical ventilation at any given time following admission to the neonatal unit (those who received only ventilation breaths for stabilisation at birth are not included), *** Defined as convulsions, intraventricular haemorrhage, periventricular leukomalacia, hypoxic ischaemic encephalopathy **** Defined as fractured clavicle or humerus or fracture at any other site, brachial plexus paralysis, and subgaleal haematoma. ***** Defined for head and craniofacial structures: anencephaly, encephalocele, holoprosencephaly, hydrocephaly, microphthalmia, anophthalmia, colobomas, microtia, cleft lip, cleft palate, severe micrognathia, macro- and macroglossia. Neck: cystic hygroma. Chest: pectus excavatum, absent or hypoplastic clavicles. Back: meningomyelocele, spina bifida. Abdomen: omphalocele, gastroschisis. Genitalia: ambiguous genitalia. Extremities: absent or limb deficiencies, polydactyly, complete syndactyly, polysyndactyly, absent digits, ectrodactyly. Cardiovascular and great vessels: tetralogy of Fallot, truncus arteriosus, hypoplastic left heart, ventricular or atrial septal defect, transposition of the great vessels, interrupted aortic arch type B, total anomaly of pulmonary venous return, hypoplasia or coarctation of the aorta. ****** early-onset and/or late-onset sepsis.

**Table 3:** Maternal stress, depression, anxiety, and participation in care: means, SDs, medians, and IQRs.

	Mean	SD	Median	25th Centile	75th Centile	Min	Max
SOL score	3.34	0.71	3.36	2.95	3.80	1	4.9
EPDS score	14.76	5.41	15	11	18	0	29
STAI state anxiety score	45.52	8.42	46	41.5	50.50	20	73
STAI trait anxiety score	45.79	3.23	47	45	48	35	57
IPP-NICU score	13.81	4.95	13	11	16.50	2	29

(Abbreviations: SOL Score—stress occurrence level; EPDS—Edinburgh Postnatal Depression Scale; STAI—State–Trait Anxiety Inventory; IPP-NICU—Index of Parental Participation in Neonatal Intensive Care Unit; SD—standard deviation).

**Table 4:** Logistic regression models including potential confounders for maternal stress.

Variable		SOL < 3 *n (*%)	SOL ≥ 3 *n* (%)	OR (Univariable) 95% CI, *p*-Value	aOR (Multivariable) 95% CI, *p*-Value
Ethnicity	Sinhala	47 (23.6)	152 (76.4)	-	-
	Tamil	20 (45.5)	24 (54.5)	0.37 (0.19–0.73, *p* = 0.004)	0.46 (0.21–1.03, *p* = 0.056)
	Muslim	13 (22.8)	44 (77.2)	1.05 (0.53–2.17, *p* = 0.899)	1.28 (0.58–2.98, *p* = 0.549)
Maternal age	Mean ± SD	29.4 ± 5.3	30.0 ± 6.0	1.02 (0.97–1.07, *p* = 0.412)	1.02 (0.97–1.08, *p* = 0.413)
Maternal occupation	Un-employed	71 (29.1)	173 (70.9)	-	-
	Working	9 (16.1)	47 (83.9)	2.14 (1.04–4.88, *p* = 0.051)	2.24 (0.97–5.77, *p* = 0.073)
APGAR at 5 minutes	Mean ± SD	7.6 ± 2.1	7.6 ± 2.3	1.00 (0.89–1.12, *p* = 0.999)	0.98 (0.84–1.13, *p* = 0.774)
Weight of the neonate	Mean ± SD	2275.5 ± 878.7	2224.9 ± 889.9	1.00 (1.00–1.00, *p* = 0.668)	1.00 (1.00–1.00, *p* = 0.186)
Gestational age in weeks	<27	2 (18.2)	9 (81.8)	-	-
	27–32	16 (27.1)	43 (72.9)	0.60 (0.09–2.64, *p* = 0.537)	0.64 (0.03–4.89, *p* = 0.708)
	32–37	20 (21.5)	73 (78.5)	0.81 (0.12–3.47, *p* = 0.799)	0.89 (0.04–7.55, *p* = 0.925)
	>37	42 (30.7)	95 (69.3)	0.50 (0.07–2.06, *p* = 0.392)	0.37 (0.02–3.80, *p* = 0.446)
Ventilated	No	23 (23.0)	77 (77.0)	-	-
	Yes	57 (28.5)	143(71.5)	0.75 (0.42–1.30, *p* = 0.311)	0.62 (0.29–1.29, *p* = 0.209)
Admitted to	NICU	59 (25.1)	176 (74.9)	-	-
	Semi-Intensive	21 (32.3)	44 (67.7)	0.70 (0.39–1.29, *p* = 0.247)	0.52 (0.25–1.07, *p* = 0.071)
Respiratory distress	No	51 (27.0)	138 (73.0)	-	-
	Yes	29 (26.1)	82 (73.9)	1.04 (0.62–1.79, *p* = 0.871)	1.66 (0.84–3.26, *p* = 0.14)
Sepsis	No	44 (23.2)	146 (76.8)	-	-
	Yes	29 (26.1)	82 (73.9)	1.04 (0.62–1.79, *p* = 0.871)	1.66 (0.84–3.26, *p* = 0.140)
Total days stayed in the hospital	<11 days	39 (26.4)	109 (73.6)	-	-
	≥11 days	40 (27.4)	106 (72.6)	0.95 (0.57–1.59, *p* = 0.840)	0.84 (0.42–1.68, *p* = 0.630)
IPP-NICU	<20	59 (22.6)	202 (77.4)	-	-
	≥20	21 (53.8)	18 (46.2)	0.25 (0.12–0.50, *p* < 0.001)	0.27 (0.12–0.59, *p* = 0.001)

Dependent variable: SOL equal to or more than 3. Mulivariable logistic model: number in dataframe = 300, number in model = 275, missing = 25, AIC = 311.2, C-statistic = 0.7, H&L = Chi-sq(8) 5.13 (*p* = 0.744). (Abbreviations: SOL—stress occurrence level; NICU—neonatal intensive care unit; IPP-NICU—Index of Parental Participation in NICU).

**Table 5:** Logistic regression models including potential confounders for maternal depression.

Variable		EPDS < 9 *n* (%)	EPDS ≥ 9 *n (*%)	OR (Univariable) 95% CI, *p*-Value	aOR (Multivariable) 95% CI, *p*-Value
Ethnicity	Sinhala	26 (13.1)	173 (86.9)	-	-
	Tamil	8 (18.2)	36 (81.8)	0.68 (0.29–1.71, *p* = 0.378)	0.36 (0.12–1.07, *p* = 0.057)
	Muslim	5 (8.8)	52 (91.2)	1.56 (0.62–4.80, *p* = 0.384)	1.25 (0.45–4.06, *p* = 0.691)
Maternal age	Mean ± SD	29.4 ± 6.3	29.9 ± 5.8	1.02 (0.96–1.08, *p* = 0.606)	1.02 (0.95–1.09, *p* = 0.627)
Maternal occupation	Un-employed	31 (12.7)	213 (87.3)	-	-
	Working	8 (14.3)	48 (85.7)	0.87 (0.39–2.15, *p* = 0.751)	1.11 (0.43–3.17, *p* = 0.835)
APGAR at 5 min	Mean ± SD	7.8 ± 2.3	7.5 ± 2.3	0.95 (0.79–1.11, *p* = 0.526)	0.92 (0.74–1.11, *p* = 0.387)
Weight of the neonate	Mean ± SD	2340.4 ± 941.0	2222.8 ± 878.1	1.00 (1.00–1.00, *p* = 0.445)	1.00 (1.00–1.00, *p* = 0.670)
Gestational age in weeks	<27	3 (27.3)	8 (72.7)	-	-
	27–32	10 (16.9)	49 (83.1)	1.84 (0.36–7.71, *p* = 0.424)	1.85 (0.09–15.30, *p* = 0.608)
	32–37	6 (6.5)	87 (93.5)	5.44 (1.00–25.29, *p* = 0.034)	9.80(0.39–118.59, *p* = 0.090)
	>37	20 (14.6)	117 (85.4)	2.19 (0.45–8.34, *p* = 0.275)	3.59 (0.12–56.62, *p* = 0.386)
Ventilated	No	15 (15.0)	85 (85.0)	-	-
	Yes	24 (12.0)	176 (88.0)	1.29 (0.63–2.57, *p* = 0.467)	1.08 (0.42–2.71, *p* = 0.876)
Admitted to	NICU	25 (10.6)	210 (89.4)	-	-
	Semi-intensive	14 (21.5)	51 (78.5)	0.43 (0.21–0.91, *p* = 0.023)	0.29 (0.12–0.68, *p* = 0.004)
Respiratory distress	No	9 (10.0)	81 (90.0)	-	-
	Yes	30 (14.3)	180 (85.7)	0.67 (0.29–1.42, *p* = 0.314)	0.34 (0.11–0.90, *p* = 0.039)
Sepsis	No	28 (14.7)	162 (85.3)	-	-
	Yes	11 (10.0)	99 (90.0)	1.56 (0.76–3.39, *p* = 0.242)	1.58 (0.72–3.67, *p* = 0.271)
Total days stayed in the hospital	<11 days	18 (12.2)	130 (87.8)	-	-
	≥days	20 (13.6)	127 (86.4)	0.88 (0.44–1.74, *p* = 0.712)	0.69 (0.28–1.72, *p* = 0.431)
IPP-NICU	<20	33 (12.6)	228 (87.4)	-	-
	≥20	6 (15.4)	33 (84.6)	0.80 (0.33–2.24, *p* = 0.636)	0.76 (0.27–2.55, *p* = 0.633)

Dependent variable: EPDS ≥ 9. Multivariable logistic model: number in dataframe = 300, number in model = 275, missing = 25, AIC = 217.2, C-statistic = 0.736, H&L = Chi-sq(8) 4.17 (*p* = 0.842) (Abbreviations: EPDS—Edinburgh Postnatal Depression Scale, NICU—neonatal intensive care unit; IPP-NICU—Index of Parental Participation in NICU).

**Table 6:** Logistic regression models including potential confounders for state anxiety.

		STAI Y1 ≤40 *n* (%)	STAI Y1 >40 *n* (%)	OR (Univariable) 95% CI, *p*-Value	aOR (Multivariable) 95% CI, *p*-Value
Ethnicity	Sinhala	33 (16.6)	166 (83.4)	-	-
	Tamil	16 (36.4)	28 (63.6)	0.35 (0.17–0.72, *p* = 0.004)	0.27 (0.12–0.63, *p* = 0.002)
	Muslim	18 (31.6)	39 (68.4)	0.43 (0.22–0.85, *p* = 0.014)	0.34 (0.16–0.73, *p* = 0.005)
Maternal age	Mean ± SD	29.1 ± 6.2	30.0 ± 5.7	1.03 (0.98–1.08, *p* = 0.256)	1.02 (0.97–1.08, *p* = 0.509)
Maternal occupation	Un-employed	58 (23.8)	186 (76.2)	-	-
	Working	9 (16.1)	47 (83.9)	1.63 (0.78–3.73, *p* = 0.216)	1.74 (0.76–4.33, *p* = 0.209)
APGAR at 5 Minutes	Mean ± SD	7.6 ± 2.1	7.6 ± 2.3	0.99 (0.87–1.11, *p* = 0.869)	0.90 (0.76–1.04, *p* = 0.153)
Weight of the neonate	Mean ± SD	2208.9 ± 840.7	2246.6 ± 899.9	1.00 (1.00–1.00, *p* = 0.762)	1.00 (1.00–1.00, *p* = 0.467)
Gestational age in weeks	<27	3 (27.3)	8 (72.7)	-	-
	27–32	16 (27.1)	43 (72.9)	1.01 (0.20–3.99, *p* = 0.992)	0.74 (0.04–5.54, *p* = 0.798)
	32–37	15 (16.1)	78 (83.9)	1.95 (0.39–7.66, *p* = 0.362)	1.66 (0.08–14.31, *p* = 0.676)
	>37	33 (24.1)	104 (75.9)	1.18 (0.25–4.35, *p* = 0.813)	0.71 (0.03–7.61, *p* = 0.797)
Ventilated	No	18 (18.0)	82 (82.0)	-	-
	Yes	49 (24.5)	151 (75.5)	0.68 (0.36–1.22, *p* = 0.204)	0.63 (0.29–1.35, *p* = 0.239)
Admitted to	NICU	53 (22.6)	182 (77.4)	-	-
	Semi- intensive	14 (21.5)	51 (78.5)	1.06 (0.56–2.13, *p* = 0.862)	0.82 (0.38–1.82, *p* = 0.618)
Any respiratory distress	No	19 (21.1)	71 (78.9)	-	-
	Yes	48 (22.9)	162 (77.1)	0.90 (0.49–1.63, *p* = 0.739)	0.76 (0.35–1.55, *p* = 0.454)
Sepsis	No	42 (22.1)	148 (77.9)	-	-
	Yes	25 (22.7)	85 (77.3)	0.96 (0.55–1.71, *p* = 0.901)	1.01 (0.54–1.94, *p* = 0.964)
Total days stayed in the hospital	<11 days	26 (17.6)	122 (82.4)	-	-
	≥days	38 (25.9)	109 (74.1)	0.61 (0.35–1.07, *p* = 0.86)	0.53 (0.25–1.07, *p* = 0.079)
IPP-NICU	<20	52 (19.9)	209 (80.1)	-	-
	≥20	15 (38.5)	24 (61.5)	0.40 (0.20–0.83, *p* = 0.011)	0.32 (0.14–0.72, *p* = 0.005)

Dependent variable: STAI state (Y1) > 40. Multivariable logistic model: number in dataframe = 300, number in model = 275, missing = 25, AIC = 297.3, C-statistic = 0.726, H&L = Chi-sq(8) 10.08 (*p* = 0.260) (Abbreviations: STAI—State–Trait Anxiety Inventory; NICU—neonatal intensive care unit; IPP-NICU—Index of Parental Participation in NICU).

## Data Availability

The raw data supporting the conclusions of this article will be made available by the authors on request. The data are not publicly available due to ethical and privacy restrictions.

## Supplementary Materials

The following supporting information can be downloaded at https://www.mdpi.com/article/10.3390/children13020247/s1. Supplemental Table S1: STROBE Statement—checklist of items that should be included in reports of observational studies; Supplemental Table S2: Maternal stress: subscores; Supplemental Table S3: Maternal participation in care in the neonatal unit-subscores; Supplemental Table S4: Maternal participation in care in the neonatal unit:subscores.

## Abbreviations

The following abbreviations are used in this manuscript:

NICU	Neonatal intensive care unit
EPiNICU	Empowering parents in the NICU
PSS:NICU	Parental Stressor Scale
SOL	Stress occurrence level
OSL	Overall stress level
EPDS	Edinburgh Postnatal Depression Scale
STAI	State- Trait Anxiety Inventory
STAI y1	State anxiety
STAI y2	Trait anxiety
IPP-NICU	Index of Parental Participation
OR	Odds ratio
aOR	Adjusted odds ratio
CI	Confidence intervals
IQR	Interquartile range
SD	Standard deviation
N	Absolute frequency
